# Heterologous production of novel and rare C_30_-carotenoids using *Planococcus* carotenoid biosynthesis genes

**DOI:** 10.1186/s12934-021-01683-3

**Published:** 2021-10-09

**Authors:** Miho Takemura, Chiharu Takagi, Mayuri Aikawa, Kanaho Araki, Seon-Kang Choi, Mitsuhiro Itaya, Kazutoshi Shindo, Norihiko Misawa

**Affiliations:** 1 Research Institute for Bioresources and Biotechnology, Ishikawa Prefectural University, 1-308 Suematsu, Nonoichi, Ishikawa 921-8836 Japan; 2 Department of Food and Nutrition, Japan Women’s University, Bunkyo-ku, Tokyo, 112-8681 Japan; 3 Department of Agriculture and Life Industry, Kangwon National University, Chuncheon-si, Gangwon-do 24341 Republic of Korea; 4 Department of Biomedical Engineering, Graduate School of Science and Technology, Shinshu University, Wakasato 4-17-1, Nagano, 380-8553 Japan

## Abstract

**Background:**

Members of the genus *Planococcus* have been revealed to utilize and degrade solvents such as aromatic hydrocarbons and alkanes, and likely to acquire tolerance to solvents. A yellow marine bacterium *Planococcus maritimus* strain iso-3 was isolated from an intertidal sediment that looked industrially polluted, from the Clyde estuary in the UK. This bacterium was found to produce a yellow acyclic carotenoid with a basic carbon 30 (C_30_) structure, which was determined to be methyl 5-glucosyl-5,6-dihydro-4,4′-diapolycopenoate. In the present study, we tried to isolate and identify genes involved in carotenoid biosynthesis from this marine bacterium, and to produce novel or rare C_30_-carotenoids with anti-oxidative activity in *Escherichia coli* by combinations of the isolated genes.

**Results:**

A carotenoid biosynthesis gene cluster was found out through sequence analysis of the *P. maritimus* genomic DNA. This cluster consisted of seven carotenoid biosynthesis candidate genes (*orf1–7*). Then, we isolated the individual genes and analyzed the functions of these genes by expressing them in *E. coli*. The results indicated that *orf2* and *orf1* encoded 4,4′-diapophytoene synthase (CrtM) and 4,4′-diapophytoene desaturase (CrtNa), respectively. Furthermore, *orf4* and *orf5* were revealed to code for hydroxydiaponeurosporene desaturase (CrtNb) and glucosyltransferase (GT), respectively. By utilizing these carotenoid biosynthesis genes, we produced five intermediate C_30_-carotenoids. Their structural determination showed that two of them were novel compounds, 5-hydroxy-5,6-dihydro-4,4′-diaponeurosporene and 5-glucosyl-5,6-dihydro-4,4′-diapolycopene, and that one rare carotenoid 5-hydroxy-5,6-dihydro-4,4′-diapolycopene is included there. Moderate singlet oxygen-quenching activities were observed in the five C_30_-carotenoids including the two novel and one rare compounds.

**Conclusions:**

The carotenoid biosynthesis genes from *P. maritimus* strain iso-3, were isolated and functionally identified. Furthermore, we were able to produce two novel and one rare C_30_-carotenoids in *E. coli*, followed by positive evaluations of their singlet oxygen-quenching activities.

**Supplementary Information:**

The online version contains supplementary material available at 10.1186/s12934-021-01683-3.

## Introduction

Carotenoids, essential pigments for photosynthesis, are known to protect cells from oxidative stress [1, 2]. In the domain bacteria, all of the photosynthetic bacteria, including cyanobacteria, as well as some of the non-photosynthetic bacteria, usually chemoheterotrophs, can produce carotenoids [3]. Among the non-photosynthetic bacteria, several species have been shown to produce acyclic carotenoids with a basic carbon 30 (C_30_) structure instead of the typical C_40_-carotenoids. Specifically, the carotenogenic-bacterial species of the phylum Firmicutes of low GC Gram-positive bacteria that have been reported to synthesize only C_30_-carotenoids include *Streptococcus faecium* [3], *Staphylococcus aureus* [4], *Bacillus firmus* [5], *Halobacillus halophilus* [6, 7], *Planococcus maritimus* [8, 9], *Sporosarcina aquimarina* [10], and *Lactobacillus plantarum* (recently reclassified to *Lactiplantibacillus plantarum*) [11]. C_30_-Carotenoids have also been found in bacterial species that belong to other phyla, such as *Rubritalea squalenifaciens* of the phylum Verrucomicrobia [12] and *Methylomonas* sp. strain 16a of the phylum Proteobacteria [13].

The biosynthesis of C_30_-carotenoids has mostly been studied in *Staphylococcus aureus* [4, 14–16]. Farnesyl diphosphate (FPP) is converted to 4,4′-diapophytoene (dehydrosqualene) by 4,4′-diapophytoene synthase (CrtM) and to 4,4′-diaponeurosporene by 4,4′-diapophytoene desaturase (CrtN) [16], which is likely to be the common biosynthetic route in all C_30_-carotenogenic bacteria. Lastly, staphyloxanthin [β-d-glucosyl 1-*O*-(4,4′-diaponeurosporen-4-oate)-6-*O*-(12-methyltetradecanoate)], which has shown to confer oxidative stress tolerance on *S. aureus* [17], is produced from 4,4′-diaponeurosporene as the final product with the mediation of four genes that encode oxidase (CrtP), dehydrogenase (AldH), glycosyltransferase (CrtQ), and acyltransferase (CrtO) [14, 15]. As for other C_30_-carotenogenic bacteria, a few studies have been published on their genes for metabolizing 4,4′-diaponeurosporene [13, 18–20].

Members of the genus *Planococcus* have been revealed to utilize and degrade solvents such as aromatic hydrocarbons and alkanes, and likely to acquire tolerance to solvents [21, 22]. A yellow marine bacterium *Planococcus maritimus* strain iso-3 was isolated from an intertidal sediment that looked industrially polluted, from the Clyde estuary in the UK [8]. Its yellow pigment was identified as methyl 5-glucosyl-5,6-dihydro-4,4′-diapolycopenoate (methyl 5-glucosyl-5,6-dihydro-apo-4,4′-lycopenoate), which showed potent antioxidative activity [8, 9]. Kim et al. [23] also reported that *Planococcus faecalis* AJ003^T^ produced a similar C_30_-carotenoid glycosyl-4,4′-diaponeurosporen-4′-ol-4-oate.

In this study, we tried to isolate genes involved in the production of the glucosyl C_30_-carotenoid from *P. maritimus* strain iso-3, to identify the functions of the isolated genes using *E. coli*, and to produce novel and rare C_30_-carotenoids with anti-oxidative activity in *E. coli* by combinations of the obtained genes.

## Materials and methods

### Bacterial strains and growth conditions

*Escherichia coli* K12 DH5α was used for DNA manipulation. *E. coli* K12 JM101(DE3) was constructed from strain JM101 using the λDE3 lysogenization kit (Merk, Darmstadt, Germany) and used for expression of the carotenoid biosynthesis genes. These *E. coli* strains and their transformants were maintained as the frozen stocks including 20% glycerol at − 80 °C, and were grown in 2 × YT medium (16 g/L of tryptone, 10 g/L of yeast extract, 5 g/L of NaCl) at 37 °C as needed. *Bacillus subtilis* strain MI112 was kindly provided from Dr. Atsuhiko Shinmyo (NAIST, Japan). This bacterium was similarly maintained as the frozen stock and routinely grown in LB medium (10 g/L of tryptone, 5 g/L of yeast extract, 10 g/L of NaCl) at 37 °C.

### DNA isolation of *Planococcus maritimus* strain iso-3

Genome DNA was prepared from *Planococcus maritimus* strain iso-3 according to a method described by Nishida et al. [24].

### Functional cloning experiments

The genome DNA was digested with BamHI, ligated with vector pGETS103, and used to transform *Bacillus subtilis* strain MI112 as described previously [25]. In generated genome library, one yellow colony appeared, which was found to contain a 7344-bp insert.

### Inverse PCR

To isolate the flanking region of the carotenoid biosynthesis gene cluster, inverse PCR was performed. One μg of genomic DNA from the *Planococcus* was digested with EcoRI. Digested DNA (0.1 μg/μL) was self-ligated with ligation solution (Takara Bio, Ohtsu, Japan). PCR was performed using 50 pg of these circular DNAs as the template in 25 μL of the reaction solution with the gene-specific primers which we designed. The amplified fragments were cloned into the pBC phagemid vector (Agilent, CA, USA), transformed into *E. coli* (DH5α) and sequenced. The primers used are summarized in Additional file 1: Table S1.

### Sequence analysis

Homology search was performed by BLAST (http://blast.ncbi.nlm.nih.gov/Blast.cgi). Amino acid alignments and phylogenetic trees were constructed using MAFFT (http://www.mafft.ccbrc.jp/).

### Cloning of the carotenoid biosynthesis genes from *P. maritimus* strain iso-3

Based on the sequences obtained, the primers containing the restriction sites were designed as shown in Additional file 1: Table S1 and the coding regions of each *orf* were amplified by PCR of the genomic DNAs.

Then PCR products were cloned into a plasmid vector and sequenced.

### Expression of the *Planococcus* carotenoid biosynthesis genes in *Escherichia coli*

Plasmids used in this study are summarized in Additional file 1: Fig. S1. Firstly, we constructed the plasmid pAC-HI which contained the *Haematococcus pluvialis IDI* (isopentenyl diphosphate isomerase) gene between the *tac* promoter (P*tac*) and the *rrnB* terminator (T*rrnB*) in the pACYC184 vector. The exogenous expression of the *IDI* gene in *E. coli* results in the increase of carotenoid production [26]. Then, the coding region of the *Planococcus orf3* was inserted into the plasmid pAC-HI. The resultant plasmid was named as pAC-HIO3. The coding regions of the *orf1*, *orf2*, *orf4* were cloned into the pAC-HIM which contained *IDI* and *Leuconostoc mesenteroides crtM,* independently. These plasmids were designated pAC-HIMO1, pAC-HIMO2, and pAC-HIMO4, respectively. The coding regions of the *orf2*, *orf4* and *orf7* were cloned into the pAC-HIMN which contained *IDI* and *L. mesenteroides crtM* and *crtN*, independently. The resultant plasmids were named as pAC-HIMNO2, pAC-HIMNO4, and pAC-HIMNO7, respectively. The *orf2* and *orf4* were also inserted into the pAC-HIMNF (same as pAC-HIMNO7) and the obtained plasmids were pAC-HIMNFO2 and pAC-HIMNFO4, respectively. The *orf5* was further inserted into the pAC-HIMNFNb (same as pAC-HIMNFO4) and this plasmid was named pAC-HIMNFNbO5 (renamed pAC-HIMNFNbG). All plasmids were independently introduced into the wild type *E. coli* (JM101 (DE3)). Each transformed *E. coli* was grown in 2YT medium at 37 °C. Next day, this culture was inoculated in a new 2YT medium (100 mL medium in 500 mL Sakaguchi flask) and cultured at 21 °C for 2 days.

The accession no. of the sequences of the plasmids, pAC-HI, pAC-HIM, pAC-HIMN, pAC-HIMNF, pAC-HIMNFNb, and pAC-HIMNFNbG are LC635748, LC647532, LC647533, LC647534, LC647535 and LC647536, respectively.

### Extraction and HPLC analysis of carotenoids from *E. coli* cells

Extraction of carotenoids from the recombinant *E. coli* was performed by the method described by Fraser et al. [27]. *E. coli* cultures were centrifuged and cell pellets were extracted with methanol (MeOH) using mixer for 5 min. Tris–HCl (50 mM, pH 7.5) and 1 M NaCl were added and mixed. Then chloroform was added to the mixture and mixed for 5 min. After centrifugation, the chloroform phase was collected and dried by centrifugal evaporation. Dried residues were re-suspended with ethyl acetate (EtOAc), and applied to HPLC with a Waters Alliance 2695–2996 (PDA) system (Waters, Milford, MA, USA). HPLC was carried out according to the method described [28] using TSKgel ODS-80Ts (4.6 × 150 mm, 5 μm; Tosoh, Tokyo, Japan). Briefly, the extract was eluted at a flow rate of 1.0 mL/min at 25 °C with solvent A [water (H_2_O)-MeOH, 5:95] for 5 min, followed by a linear gradient from solvent A to solvent B (tetrahydrofuran-MeOH, 3:7) for 5 min, and solvent B alone for 8 min. The produced carotenoids were identified by comparing both retention times and absorption spectra with those of our authentic standards. When the produced carotenoids are not compounds in our authentic standards as described in the following sections, we isolated the produced carotenoids and determined their structures using HRESI-MS (high resolution electrospray ionization-mass spectrometry) and NMR (nuclear magnetic resonance) analyses.

### Isolation of 5-hydroxy-5,6-dihydro-4,4′-diaponeurosporene (3)

The transformed *E. coli* cells carrying *crtM*, *crtNa*, and *cruF* were collected by centrifugation from 4 L culture, and extracted with 50 mL acetone-MeOH (7:2) and dichloromethane (CH_2_Cl_2_) (×2) with sonication in a stepwise manner. The combined extract (150 mL) was concentrated to small volume in vacuo, and partitioned between EtOAc/H_2_O (each 100 mL). The *n*-hexane layer evaporated to dryness (54.2 mg) and subjected to silica gel chromatography (20 mm × 200 mm) using *n*-hexane–acetone (8:1), and fractionated by 10 mL. The fractions 15–30 containing a yellow compound [Rf 0.4 by silica gel TLC using developing solvent *n*-hexane–acetone (4:1)] were collected and concentrated to afford a yellow oil (27.1 mg). The yellow oil was applied on preparative HPLC (column: Develosil C-30-UG-5 (20 × 250 mm, Nomura Chemical, Co. Ltd, Aichi, Japan), solvent: CH_2_Cl_2_-CH_3_CN (4:6), flow rate: 8.0 mL/min, detect: PDA 250–700 nm), and a yellow peak eluted at 15.0 min was collected and concentrated to afford pure 5-hydroxy-5,6-dihydro-4,4′-diaponeurospore (**3**, 13.9 mg).

### Isolation of 5-glucosyl-5,6-dihydro-4,4′-diapolycopene (5)

The transformed *E. coli* cells carrying *crtM*, *crtNa*, *cruF*, *crtNb*, and *GT* were collected by centrifugation from 4 L culture, and extracted with acetone-MeOH (7:2) and CH_2_Cl_2_-MeOH (1:1) (×2) with sonication in a stepwise manner. The extract (150 mL) was concentrated to small volume in vacuo, and partitioned between EtOAc/H_2_O (each 100 mL). The EtOAc layer evaporated to dryness (243.9 mg) and subjected to silica gel chromatography (25 mm × 190 mm) using CH_2_Cl_2_-MeOH (10:1) and fractionated by 10 mL. The fractions 13–15 containing an UV compound [Rf 0.36 by silica gel TLC using developing solvent CH_2_Cl_2_-MeOH (10:1)] to afford a yellow oil (11.2 mg). The yellow oil was applied on preparative HPLC [column: Develosil C-30-UG-5 (20 × 250 mm), solvent: CH_2_Cl_2_-MeOH (1:4), flow rate: 8.0 mL/min, detect: PDA 250–700 nm], and a yellow peak eluted at 17.0 min was collected and concentrated to afford pure 5-glucosyl-5,6-dihydro-4,4′-diapolycopene (**5**, 1.4 mg).

### Singlet oxygen-quenching activity

For the measurement of singlet oxygen-quenching activity, 80 μL of 25 μM methylene blue and 100 μL of 0.24 M linoleic acid, with or without 40 μL of carotenoid (final concentration, 1–100 μM; each dissolved in ethanol), were added to 5 mL glass test tubes. The tubes were mixed well and were illuminated at 7000 lx at 22 °C for 3 h in a styrofoam box. Then, 50 μL of the reaction mixture was removed and diluted to 1.5 mL with ethanol, and OD_235_ was measured to estimate the formation of conjugated dienes [29]. The OD_235_ in the absence of carotenoids was measured as negative control [no singlet oxygen (^1^O_2_)-quenching activity], and the ^1^O_2_-quenching activity of carotenoids was calculated from OD_235_ in the presence of carotenoids relative to this reference value. The activity was indicated as the IC_50_ value, which represents the concentration at which 50% inhibition was observed.

## Results

### Isolation of the carotenoid biosynthesis gene cluster from *P. maritimus* strain iso-3

**Fig. 1:**
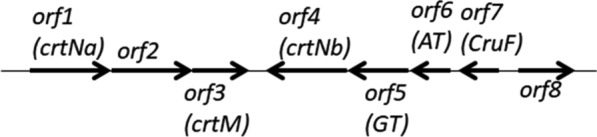
Gene organization of the DNA fragments obtained from the *P. maritimus* strain iso-3

A yellow colony including a 7.3-kb genomic insert from *P. maritimus* strain iso-3 was obtained by functional cloning experiments using *Bacillus subtilis* as the host. Sequence analysis of this insert showed the presence of seven open reading frames (*orfs*), named *orfs* 2–8 (Fig. 1). Six *orfs*, *orf2*–*7*, were predicted to be involved in carotenoid biosynthesis, and the remaining *orf*, *orf8*, was unlikely to be involved in it. When the 7.3-kb (7344-bp) DNA fragment was expressed in *E. coli*, the recombinant cells did not become yellow. This DNA fragment contains the 4,4′-diapophytoene synthase (*crtM*) gene, but it does not include the 4,4′-diapophytoene desaturase (*crtNa*) gene, as shown later. It is thus likely that the recombinant *E. coli* produced only the colorless 4,4′-diapophytoene. On the other hand, the *B. subtilis* used as the host retained 4,4′-diapophytoene desaturase activity; therefore, the recombinant *Bacillus* cells turned yellow.

The complete genome sequences of several *Planococcus*, such as *P. plakortidis* (NZ_CP016539.2), *P. faecalis* (NZ_CP019401.1), and *P. halocrytophilus* (NZ_CP016537.2), have been determined. Each of the sequences contains a similar carotenoid biosynthesis gene cluster that includes one more carotenoid biosynthesis gene other than the *orfs2–8.* Then, we performed inverse PCR to isolate the flanking region of *orfs2–8*. As a result, the 2.1-kb fragment adjacent to *orf2* was isolated and the additional *orf*, named *orf1*, was found. In total, a 9.4-kb genomic DNA was analyzed to find out that eight *orfs* existed (Fig. 1). The accession no. of the sequences of this genomic DNA is LC620265.

### Sequence analysis of the carotenoid biosynthesis gene candidates

**Fig. 2:**
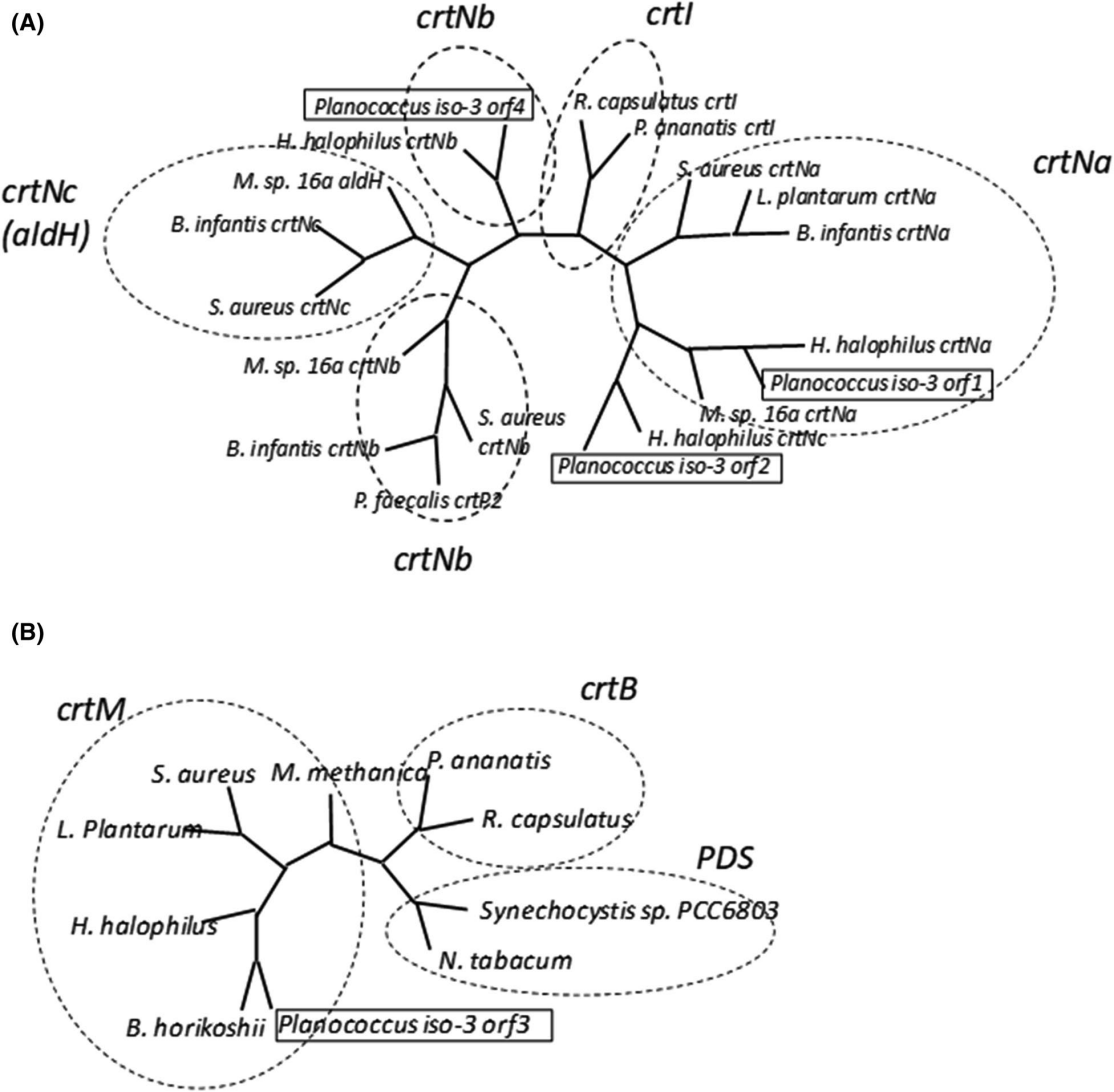
Phylogenetic trees of the *crtN*-related and *crtM*-related genes. Amino acid alignments and phylogenetic trees were constructed using MAFFT (http://www.mafft.ccbrc.jp/). **A** Phylogenetic tree of the *crtN*-related genes. The accession numbers of these sequences are shown in Additional file 1: Table S2. Orf1 and orf4 belonged to the crtNa and crtNb groups, respectively. **B** Phylogenetic tree of the *crtM*-related genes. The accession numbers of these sequences are shown in Additional file 1: Table S3. Orf3 belonged to the crtM group

Next, we performed a sequence analysis of *orf1–8*. Homology searches suggested that seven *orfs*, *orf1–7*, were involved in carotenoid biosynthesis, but not *orf8*, which was homologous to the genes encoding the MurR/RpiR family of transcriptional regulators. The predicted amino acid sequences of Orf1, Orf2, and Orf4 were homologous to that of diapophytoene desaturase. Orf1 was most homologous, displaying approximately 61% identity to the *H. halophilus* CrtNa. Whereas Orf2 and Orf4 were homologous to the *H. halophilus* CrtNc and CrtNb, displaying about 50 and 52% identity, respectively (Fig. 2A). On the other hand, Orf3 showed homology to diapophytoene synthase (CrtM) (Fig. 2B). Meanwhile, Orf5 and Orf6 were similar to glycosyltransferase and acyltransferase, respectively. Lastly, Orf7 was homologous to the genes that were annotated as carotenoid biosynthesis genes but with unknown functions. However, we found that the Orf7 was slightly similar, at 22% identity, to CruF (1′-hydroxylase).

### Orf3 acts as a diapophytoene synthase

**Fig. 3:**
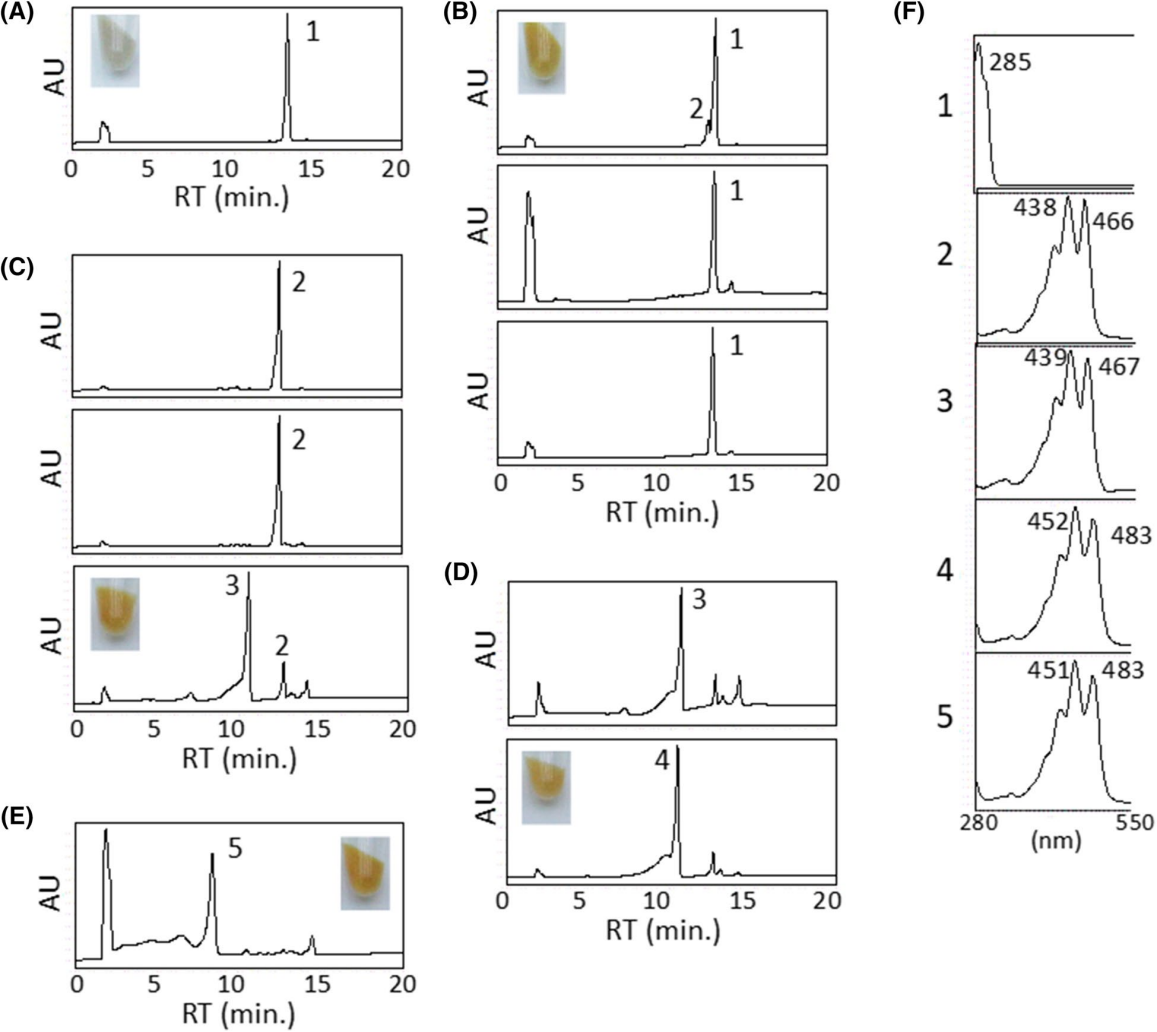
Functional analysis of *orfs*. **A** HPLC chromatogram of cell extracts of the *E. coli* expressing pAC-HIO3. **B** HPLC chromatograms of the extracts of the *E. coli* expressing the plasmids pAC-HIMO1 (upper), pAC-HIMO2 (middle), and pAC-HIMO4 (lower). **C** HPLC chromatograms of the extracts of the *E. coli* expressing the plasmids pAC-HIMNO2 (upper), pAC-HIMNO4 (middle), and pAC-HIMNO7 (lower). **D** HPLC chromatograms of the extracts of the *E. coli* expressing the plasmids pAC-HIMNFO2 (upper) and pAC-HIMNFO4 (lower). **E** HPLC chromatogram of the extracts of the *E. coli* expressing the plasmid pAC-HIMNFNbO5. **F** The spectra correspond to the peak 1: 4,4′-diapophytoene, peak 2: 4,4′-diaponeurosporene, peak 3: 5-hydroxy-5,6-dihydro-4,4′-diaponeurosporene, peak 4: 5-hydroxy-5,6-dihydro-4,4′-diapolycopene, peak 5: 5-glucosyl-5,6-dihydro-4,4′-diapolycopene. Inset show the bacterial cell pelette of each recombinant *E. coli*

**Fig. 4:**
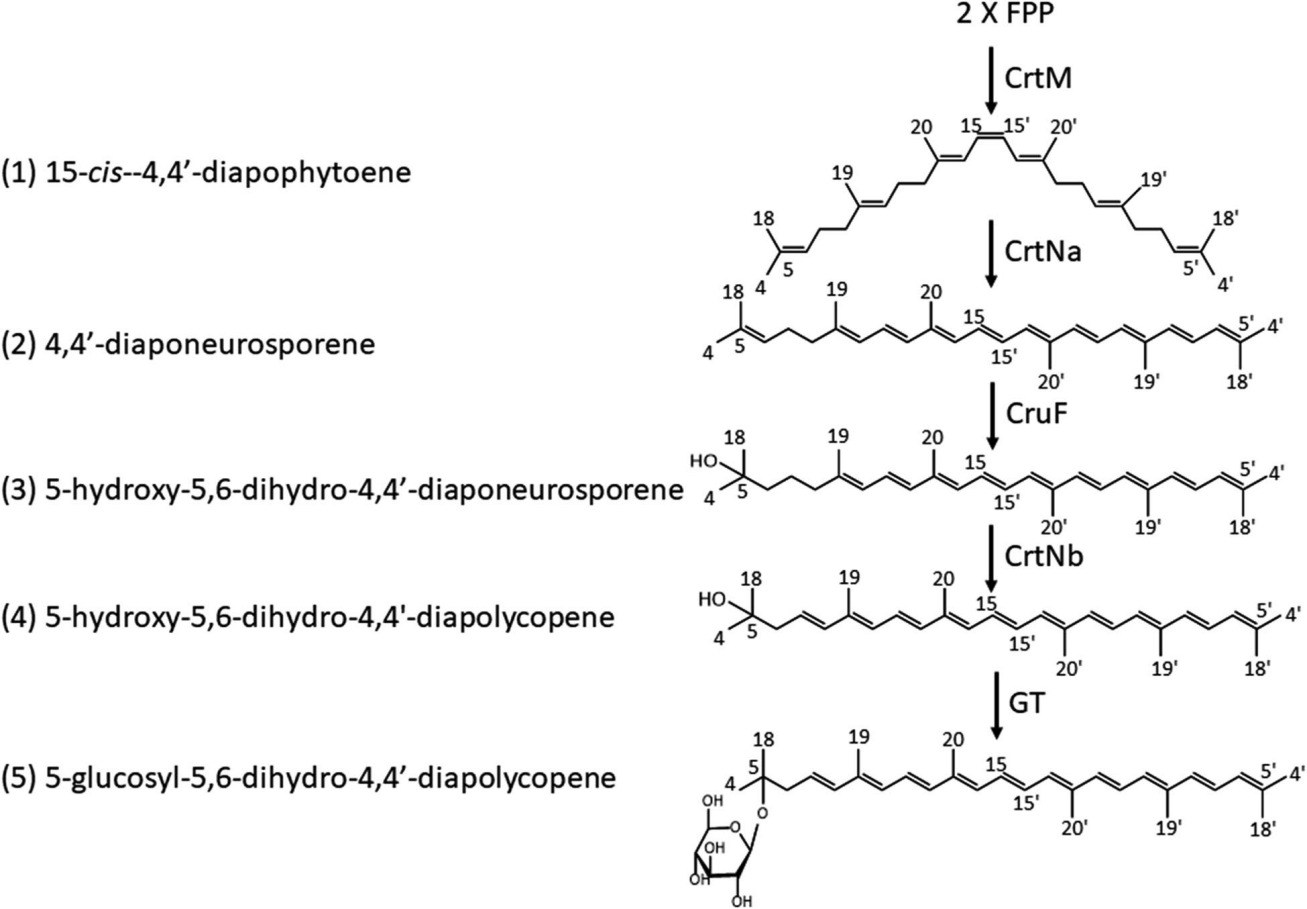
Carotenoid biosynthetic pathway in *Planococcus* strain iso-3

To investigate the function of *orf3* which was homologous to diapophytoene synthase, we made the plasmid pAC-HIO3 which included *H. pluvialis IDI* and *orf3*, and introduced it into the *E. coli* (JM101(DE3)). Wild type *E. coli* cannot produce diapophytoene from farnesyl pyrophosphate (FPP). On the other hand, a new peak (named **1**) was observed in the *E. coli* expressing pAC-HIO3 with *IDI* and *orf3* (Fig. 3A). Then, the produced compound **1** was purified from the cells via acetone-MeOH extraction, *n*-hexane/90% MeOH partition, and silica gel column chromatography and analyzed by ESI–MS (+), ^1^H, and ^13^C NMR spectra. From these spectra, the produced compound was identified as 15-*cis*-4,4′-diapophytoene (carotenoid **1**) (Fig. 4) [30]. Thus, *orf3* was confirmed to encode a 15-*cis*-4,4′-diapophytoene synthase (CrtM).

### Orf1 acts as a diapophytoene desaturase

Because *orf1*, *orf2*, and *orf4* were found to be homologous to phytoene desaturase, the catalytic activities of these Orfs were examined by constructing the plasmids pAC-HIMO1, pAC-HIMO2, and pAC-HIMO4 containing *H. pluvialis IDI*, *Leuconostoc mesenteroides crtM*, and each *orf*. The recombinant *E. coli* expressing pAC-HIMO1 generated a new carotenoid product, as indicated by the new peak (**2**) (Fig. 3B). On the other hand, the introductions of pAC-HIMO2 and pAC-HIOM4 did not affect the carotenoid profiles (Fig. 3B) We cultured the *E. coli* expressing pAC-HIMO1 and purified the carotenoid **2** using the same process described in the previous section. The ESI–MS (+), ^1^H, and ^13^C NMR spectral analyses indicated carotenoid **2** as 4,4′-diaponeurosporene (Fig. 4) [31]. These results suggest that the *orf1*, but not *orf2* or *orf4*, acts as a diapophytoene desaturase (CrtNa). However, the activity of Orf1 was low in *E. coli*; therefore, we use *L. mesenteroides crtN* for further experiments.

### Orf7 acts as a diaponeurosporene hydratase

The synthetic pathway from FPP to diaponeurosporene is thought to be general, but the reaction after diaponeurosporene varies. So, we investigated the activities of Orf2, Orf4, and Orf7 by constructing the plasmids, pAC-HIMNO2, pAC-HIMNO4, and pAC-HIMNO7, which contained *H. pluvialis IDI*, *L. mesenteroides crtM*, *L. mesenteroides crtN* and each *orf*. When pAC-HIMNO2 or pAC-HIMNO4 was introduced into the *E. coli*, it only produced 4,4′-diaponeurosporene (Fig. 3C). In the *E. coli* expressing pAC-HIMNO7 (*orf7*), a new peak (**3**) was detected, which could not be identified using our standards, in addition to 4,4′-diaponeurosporene (Fig. 3C). Thus, we cultured this recombinant *E. coli* and purified the new carotenoid **3 (**13.9 mg) as orange powder as described in the Material and Methods section. HRESI-MS (+) analysis (C_30_H_44_ONa *m/z* 443.33015 (M+Na)^+^, calcd. for 443.32896) determined the molecular formula of peak **3** as C_30_H_44_O (4,4′-diaponeurosporene (**2** + H_2_O). Detailed 1D NMR (^1^H and ^13^C) and 2D NMR [^1^H–^1^H COSY (correlation spectroscopy), HMQC (heteronuclear multiple quantum correlation), and HMBC (heteronuclear multiple bond correlation)] spectral analyses of **3** showed the ^1^H and ^13^C signals of carotenoids **2** and **3** to be quite similar except the ^5,6^Δ signals in carotenoid **2** and one methylene [H-6 (δ_H_ 1.42–1.49) and C-6 (δ_C_ 43.4)] and one sp^3^ quaternary carbon [C-5 (δ_C_ 80.0) signals in carotenoid **3** (Additional file 1: Fig. S2)]. Finally, the ^1^H–^1^H vicinal spin network of H-6–H-8 observed in ^1^H–^1^H COSY, and ^1^H–^13^C long-range couplings from H-4 and H-18 to C-5 and C-6 observed in HMBC indicated the structure of carotenoid **3** as 5-hydroxy-5,6-dihydro-4,4′-diaponeurosporene (Fig. 4). Carotenoid **3** was a new one according to the CAS database. The assigned ^1^H and ^13^C signals in **3** were listed in Table 1. These results indicate that *orf7* encodes a diaponeurosporene hydratase; thus, we named *orf7* as *cruF*, encoding a γ-carotene 1′ hydratase involved in myxoxanthophyll biosynthesis in *Synechococcus*. We also renamed the plasmid pAC-HIMNO7 to pAC-HIMNF.

**Table 1.** ^1^H and ^13^C NMR data for 5-hydroxy-5,6-dihydro-4,4′-diaponeurosporene (**3**) in CDCl_3_ and 5-glucosyl-5,6-dihydro-4,4′-diapolycopene (**5**) in DMSO-*d*_*6*_

Position	**3**		**5**	
	δ_H_	δ_C_	δ_H_	δ_C_
4	1.22 (3H, s)	29.3 (q)	1.14 (3H, s)	26.4 (q)
5		80.0 (s)		76.8 (s)
6	1.42–1.49 (2H)	43.4 (t)	2.32 (2H)	45.1 (t)
7	1.46–1.52 (2H)	22.6 (q)	5.88 (1H, m)	126.9 (d)
8	2.11 (1H, dd, 7.0, 7.3)	40.5 (t)	6.14 (1H, d, 16.4)	137.0 (d)
9		139.5 (s)		135.6 (s)
10	5.96 (1H, d, 8.9)	126.0 (d)	6.13 (1H, d, 10.0)	130.3 (d)
11	6.49 (1H, dd, 8.9, 14.9)	125.0 (d)	6.62 (1H, dd, 10.0, 15.9)	125.5 (d)
12	6.24 (1H, d, 14.9)	135.4 (d)	6.36 (1H, d, 15.9)	137.4 (d)
13		136.0 (s)		136.1 (s)^b^
14	6.19 (1H, m)	131.5 (d)	6.32 (1H, m)	132.8 (d)^c^
15	6.60 (1H, m)	130.1 (d)	6.69 (1H, m)	130.5 (d)
18	1.22 (3H, s)	29.3 (q)	1.14 (3H, s)	26.8 (q)
19	1.82 (3H, s)	16.8 (q)	1.87 (3H, s)	13.0 (q)
20	1.96 (3H, s)	12.8 (q)^a^	1.92 (3H, s)	12.7 (q)^d^
4′	1.82 (3H, s)	18.6 (q)	1.78 (3H, s)	26.2 (q)
5′		136.0 (s)		135.6 (s)
6′	5.93 (1H, d, 9.6)	126.1 (d)	5.91 (1H, d, 10.0)	126.9 (d)
7′	6.47 (1H, dd, 9.6, 15.2)	124.8 (d)	6.46 (1H, dd, 10.0, 14.9)	125.1 (d)
8′	6.22 (1H, d, 15.2)	135.0 (d)	6.22 (1H, d, 14.9)	169.1 (s)
9′		136.4 (s)		136.4 (s)^b^
10′	6.25 (1H, d, 11.8)	132.6 (d)	6.21 (1H, d, 10.0)	131.6 (d)
11′	6.61 (1H, dd, 11.8, 14.9)	124.9 (d)	6.64 (1H, dd, 10.0, 15.2)	125.4 (d)
12′	6.35 (1H, d, 14.9)	137.3 (d)	6.37 (1H, d, 15.2)	137.2 (d)
13′		136.2 (s)		136.4 (s)^b^
14′	6.19 (1H, m)	131.5 (d)	6.32 (1H, m)	132.6 (d)^c^
15′	6.60 (1H, m)	129.5 (d)	6.69 (1H, m)	130.5 (d)
18′	1.82 (3H, s)	26.3 (q)	1.78 (3H, s)	18.6 (q)
19′	1.95 (3H, s)	12.8 (q)^a^	1.92 (3H, s)	12.7 (q)^d^
20′	1.96 (3H, s)	12.9 (q)^a^	1.92 (3H, s)	13.0 (q)^d^
1″			4.32 (1H, d, 7.9)	97.4 (d)
2″			2.89 (1H, dd, 7.9, 8.0)	73.7 (d)
3″			3.15 (1H, dd, 8.0, 8.8)	76.8 (d)
4″			3.05 (1H, m)	70.4 (d)
5″			3.05 (1H, m)	71.6 (d)
6″			3.40 (1H, m), 3.61 (1H, m)	61.4 (t)

^a, b, c, d^Interchangeable

### Orf4 acts as a 5-hydroxy-5,6-dihydro-4,4′ diaponeurosporene desaturase

Since the subsequent reaction of carotenoid synthesis was expected to be desaturation, we constructed the plasmids pAC-HIMNFO2 and pAC-HIMNFO4. When pAC-HIMNFO2 was introduced into *E. coli*, only the production of 5-hydroxy-5,6-dihydro-4,4′-diaponeurosporene was detected (Fig. 3D). But in the *E. coli* expressing pAC-HIMNFO4, a new peak (**4**) was observed near the peak of 5-hydroxy-5,6-dihydro-4,4′-diaponeurosporene (Fig. 3D). The new compound **4** was identified as 5-hydroxy-5,6-dihydro-4,4′-diapolycopene, which was a rare compound in nature, using HPLC analysis with the standards (Fig. 4) [6, 7]. This data suggested that *orf4* encodes a carotenoid desaturase; thus, we named *orf4* as *crtNb* and changed the name of the plasmid pAC-HIMNFO4 to pAC-HIMNFNb.

### Orf5 acts as a glycosyltransferase

A novel carotenoid previously isolated from *Planococcus* iso-3, methyl 5-glucosyl-5,6-dihydro-4,4′-diapolycopenate, was glycosylated. Because *orf5* showed homology to glycosyltransferase, we investigated the activity of Orf5. The introduction of the plasmid pAC-HIMNFNbO5 into *E. coli* resulted in the detection of a new peak (**5**), which could not be identified using the standards (Fig. 3E). Thus, we cultured this recombinant *E. coli* and purified the new carotenoid (**5**, 13.9 mg) as orange powder. HRESI-MS (+) analysis [C_36_H_52_O_6_Na *m/z* 603.36794 (M+Na)^+^, calcd. for 603.36616] determined the molecular formula of carotenoid **5** as C_36_H_52_O_6_ (5-hydroxy-5,6-dihydro-4,4′-diapolycopene **4** + C_6_H_12_O_6_–H_2_O). Detailed 1D NMR (^1^H and ^13^C) and 2D NMR (^1^H–^1^H COSY, HSQC, and HMBC) spectral analyses of **5** demonstrated that the structure of 5-hydroxy-5,6-dihydro-4,4′-diapolycopene (carotenoid **4**) was present also in carotenoid (**5**) and the attachment of a hexose at C-5 of carotenoid **4**. The hexose in carotenoid **5** was shown to be β-glucose because all vicinal coupling constants among H-1″ to H-5″ were large (7.9–9.1 Hz) in the ^1^H NMR spectrum of acetylated carotenoid **5** in CDCl_3_ (Additional file 1: Fig. S3). From these observations, carotenoid **5** was determined to be 5-glucosyl-5,6-dihydro-4,4′-diapolycopene (Fig. 4). Carotenoid **5** was a new compound according to the CAS database. The assigned ^1^H and ^13^C signals in **5** were listed in Table 1. These results indicate that the *orf5* encodes a glycosyltransferase and we renamed pAC-HIMNFNbO5 as pAC-HIMNFNbG.

### Singlet oxygen-quenching activity of carotenoids 1 to 5

In the previous study, we reported ^1^O_2_-quenching activity of methyl 5-glucosyl-5,6-dihydro-4,4′-diapolycopenoate [8, 9]. Thus, we evaluated ^1^O_2_-quenching activities of the intermediates, carotenoids **1**–**5** isolated in this study. These five carotenoids showed milder ^1^O_2_-quenching activities than methyl 5-glucosyl-5,6-dihydro-4,4′-diapolycopenoate (Table 2).

**Table 2:** Singlet oxygen quenching activity of the intermediate carotenoids

Carotenoid	Singlet oxygen quenching IC_50_ (μM)
15-cis-4,4′-Diapophytoene (1)	> 100
4,4′-Diaponeurosporene (2)	45
5-Hydroxy-5,6-dihydro-4,4′-diaponeurosporene (3)	56
5-Hydroxy-5,6-dihydro-4,4′-diapolycopene (4)	30^a^
5-Glucosyl-5,6-dihydro-4,4′-diapolycopene (5)	30
Methyl 5-glucosyl-5,6-dihydro-4,4′-diapolycopenoate	5.1^a^
Astaxanthin	3.7^a^

^a^Cited from the data of Shindo et al. [9]

## Discussion

Until now, there are a few studies on the genes involved in the carotenoid biosynthesis in *Planococcus*. Recently, Lee et al. [19] reported the C_30_-carotenoid biosynthesis genes of *Planococcus faecalis* AJ003^T^, which mediated glycosyl-4,4′-diaponeurossporene-4′-ol-4-oic acid. Herein, we report the carotenoid biosynthesis genes in *Planococcus maritimus* strain iso-3, which produces the rare C_30_ carotenoid, methyl 5-glucosyl-5,6-dihydro-4,4′-diapolycopenoate.

**Fig. 5:**
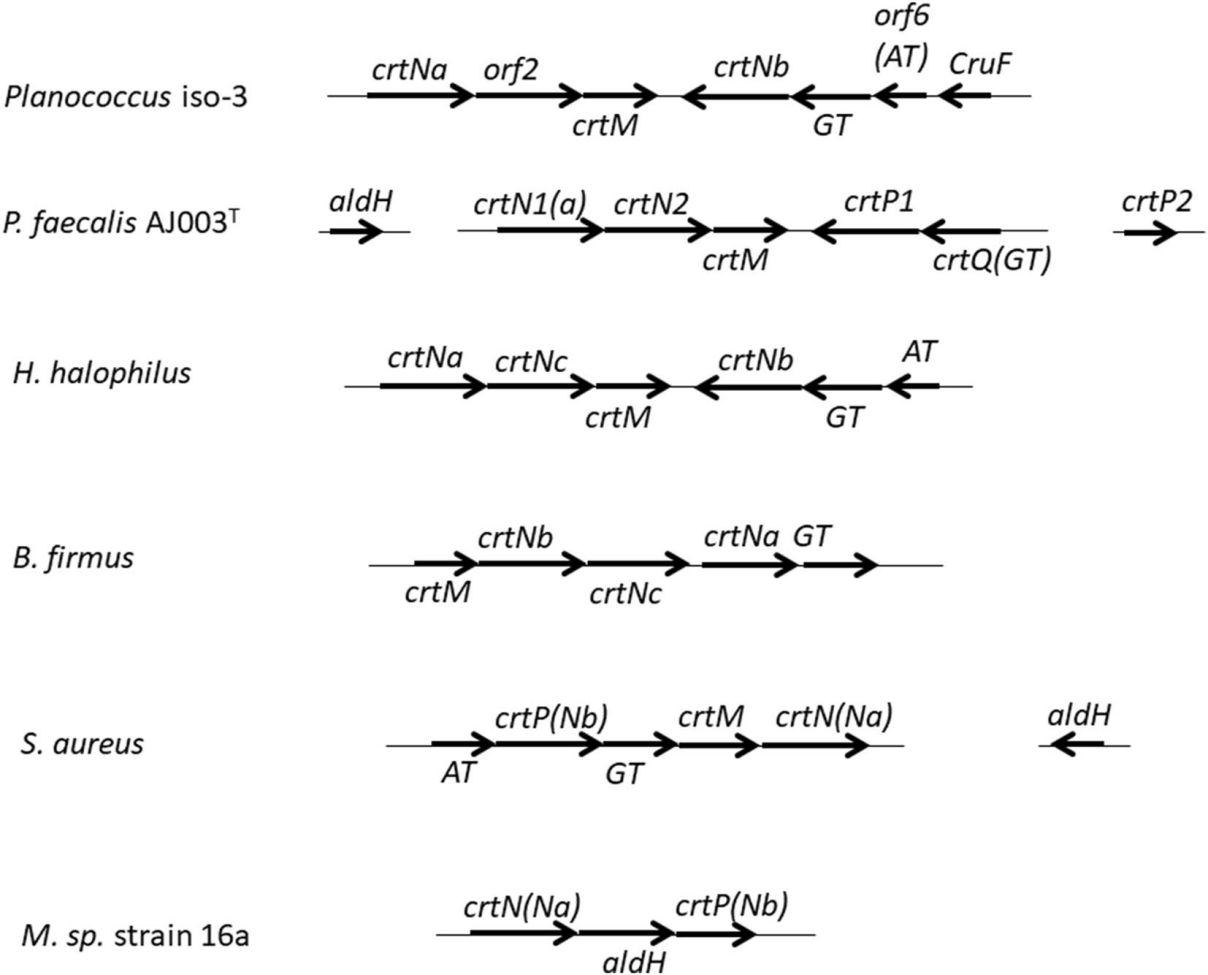
Organization of the carotenoid biosynthesis genes from C_30_ carotenoid synthesizing bacteria

The whole-genome sequences of several kinds of *Planococcus* were available in the genome database (https://www.ncbi.nlm.nih.gov/genome/). All of them, including *P. faecalis* AJ003^T^, had identical carotenoid biosynthesis gene clusters to that found in this study [19, 23]. Similar gene clusters have also been reported in *Halobacillus halophilus*, *Staphylococcus aureus*, *Bacillus indicus*, and *Bacillus firmus*, which all belong to the order of Bacillales (Fig. 5) [14, 20]. In particular, *H. halophilus* has nearly the same gene organization as *Planococcus* regarding gene order and transcriptional direction, whereas they fall into distinct families, the Bacillaceae and Planococcaceae families, respectively [18]. On the other hand, the gene organization is much different between *B. indicus* and *B. firmus* [20]. These results suggested that the ancestor of Bacillales retained the same carotenoid biosynthesis gene cluster, and during evolution, the gene organizations have been rearranged independently.

Functional analysis of the genes isolated from *P. maritimus* indicated that the *crtM* and *crtNa* genes encoded proteins with enzymatic activities as predicted from the sequence homology analysis. On the other hand, the enzyme encoded by *crtNb* was found to generate a product different from those by other *crtNb* gene products. For example, Steiger et al. [20] demonstrated that the proteins encoded by *B. indicus* and *B. firmus crtNb* functioned as aldehyde synthases for utilizing 4,4′-diapolycopene or 4,4′-diaponeurosporene. In contrast, the functions of the *crtNb* genes in *H. halophilus* and *P. fecalis* sp. Nov have not been revealed [18, 19, 32]. To discuss the evolution of *crtNb* gene, it will be required to characterize the function of various *crtNb* genes.

We also found a carotenoid 1,2-hydratase gene that showed a slight similarity to *cruF*, initially found in *Deinococcus* [33]. CruF catalyzes the reaction from γ-carotene to 1′-OH-γ-carotene and is required to produce deinoxanthin in *Deinococcus*. The homologous genes with this were present not only in the *Planococcus* genomes but also in the *Halobacillus* genomes at the same position, whereas their functions have not been elucidated. As for other-type hydratase genes, *crtC* has been reported in *Rubivivax gelatinosus* [34, 35]. However, the Orf7 did not show any similarity to the CrtC protein.

Here, the entire carotenoid biosynthetic pathway was almost elucidated using complementation analysis with *E. coli* (Fig. 4). After 15-*cis*-4,4′-diapophytoene (**1**) is synthesized, it is desaturated to 4,4′-diaponeurosporene (**2**), hydrated to produce 5-hydroxy-5,6-dihydro-4,4′-diaponeurosporene (**3**), and then desaturated to produce 5-hydroxy-5,6-dihydro-4,4′-diapolycopene (**4**). Finally, 5-glucosyl-5,6-dihydro-4,4′-diapolycopene (**5**) is produced by the glycosylation of a terminal group of 5-hydroxy-5,6-dihydro-4,4′-diapolycopene (**4**). However, according to previous reports, the final product is methyl 5-glucosyl-5,6-dihydro-apo-4,4′-lycopenoate, indicating that more reactions should occur. We found *orf6*, homologous to acyltransferase in the carotenoid gene cluster and speculated that it catalyzed the esterification. On the other hand, the genes involved in generating carotenoid carboxylic acid were not identified in the gene cluster. Indeed, the introduction of *orf2* into the *E. coli* carrying the plasmid pAC-HIMNFNb or pAC-HIMNFNbO5 did not affect the carotenoid profile (Additional file 1: Fig. S4). Lee et al. [19] reported that *crtP* and *aldH* genes coded for 4,4-diaponeurosporene oxidase and aldehyde dehydrogenase, respectively; that these genes were located away from the carotenoid gene cluster in *P. faecalis*. Thus, it is necessary to analyze the genes outside of the carotenoid synthesis gene cluster in the *Planococcus* iso-3 genome.

We evaluated the ^**1**^O_2_-quenching activities of carotenoids **1**–**5**, and compared their activities with 5-glucosyl-5,6-dihydro-4,4′-diapolycopenoate. It is very interesting that the compound produced through more advanced biosynthetic pathway shows more potent ^1^O_2_-quenching activity. *P. maritimus* strain iso-3 may have completed this biosynthetic pathway to obtain a strong singlet oxygen scavenger.

## Conclusions

We isolated seven carotenoid biosynthesis gene candidates from *P. maritimus* strain iso-3, and functionally identified five of the seven candidates as the *crtM*, *crtNa*, *crtNb*, *cruF* and *GT* genes. By utilizing these genes, we produced two novel C_30_-carotenoids, 5-hydroxy-5,6-dihydro-4,4′-diaponeurospore (**3**) and 5-glucosyl-5,6-dihydro-4,4′-diapolycopene (**5**), in addition to one rare C_30_-carotenoid 5-hydroxy-5,6-dihydro-4,4′-diapolycopene (**4**), in *E. coli*. The yield of them were estimated to be 10.8 mg/L, 2.2 mg/L and 2.6 mg/L for **3**, **4** and **5**, respectively. These compounds were shown to exhibit moderate singlet oxygen-quenching activities. In the near future, by using new carotenogenic genes, other novel and rare C_30_-carotenoids will be produced in *E. coli*.

## Supplementary Information

**Additional file 1: Figure S1.** The scheme of the constructed plasmids. **Figure S2.** NMR spectra of 5-hydroxy-5,6-dihydro-4,4′-diaponeurosporene (**3**) in CDCl_3_. **Figure S3.** NMR spectra of 5-glucosyl-5,6-dihydro-4,4′-diapolycopenene (**5**) in DMSO-*d*_6_. **Figure S4.** Functional analysis of *orf2*. **Table S1.** Primers used in this study. **Table S2.** Accession numbers of the genes used in Fig. 2A. **Table S3.** Accession numbers of the genes used in Fig. 2B.

## Data Availability

The *Planococcus* genomic sequences decided during this study are available in GenBank with the accession no. LC620265. The sequences of the plasmids, pAC-HI, pAC-HIM, pAC-HIMN, pAC-HIMNF, pAC-HIMNFNb, and pAC-HIMNFNbG are available in GenBank with the accession no. LC635748, LC647532, LC647533, LC647534, LC647535 and LC647536, respectively. Data and materials used during this study are available from the corresponding author on reasonable request.
