# Supplementary material for: Heterologous production of novel and rare C30-carotenoids using Planococcus carotenoid biosynthesis genes
Source: Microb Cell Fact. 2021 Oct 9;20:194. doi: 10.1186/s12934-021-01683-3 (PMC8502411; doi:10.1186/s12934-021-01683-3)
Supplement: Supplementary file 1 — Additional file 1: Figure S1. The scheme of the constructed plasmids. Figure S2. NMR spectra of 5-hydroxy-5,6-dihydro-4,4′-diaponeurosporene (3) in CDCl3. Figure S3. NMR spectra of 5-glucosyl-5,6-dihydro-4,4′-diapolycopenene (5) in DMSO-d6. Figure S4. Functional analysis of orf2. Table S1. Primers used in this study. Table S2. Accession numbers of the genes used in Fig. 2A. Table S3. Accession numbers of the genes used in Fig. 2B. [file 12934_2021_1683_MOESM1_ESM.docx]

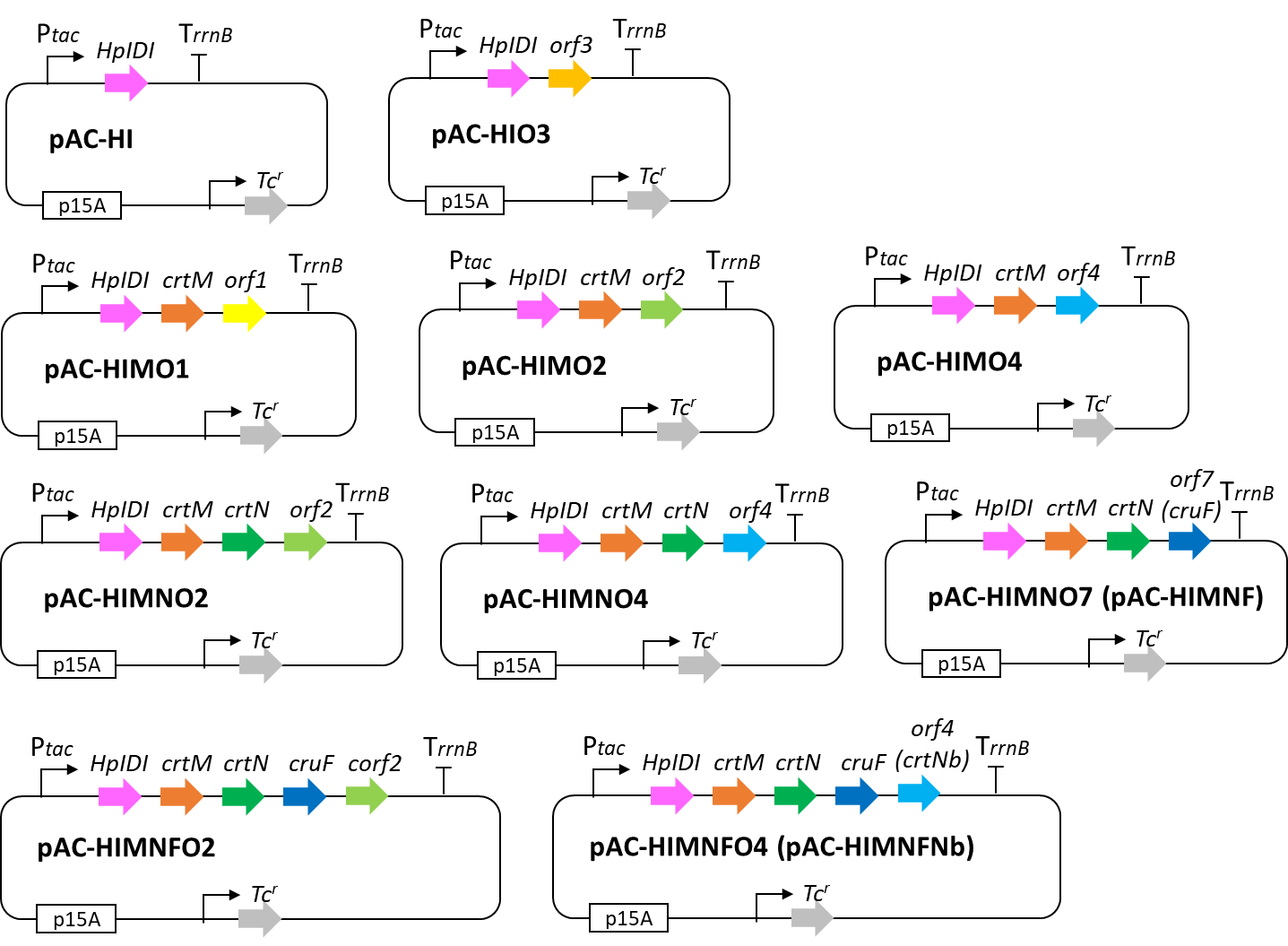

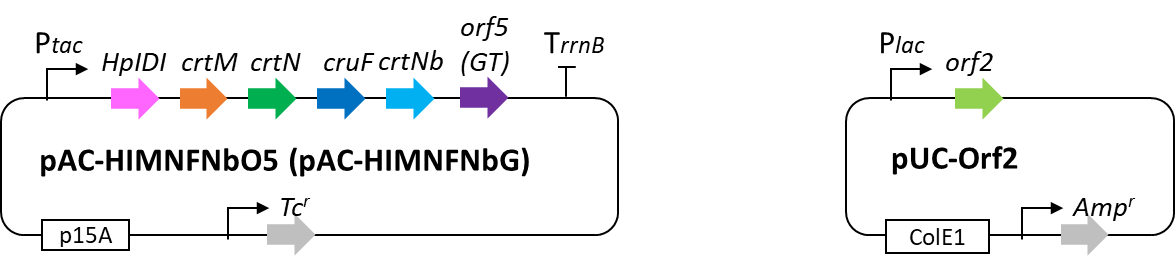


Fig. S1. The scheme of the constructed plasmids


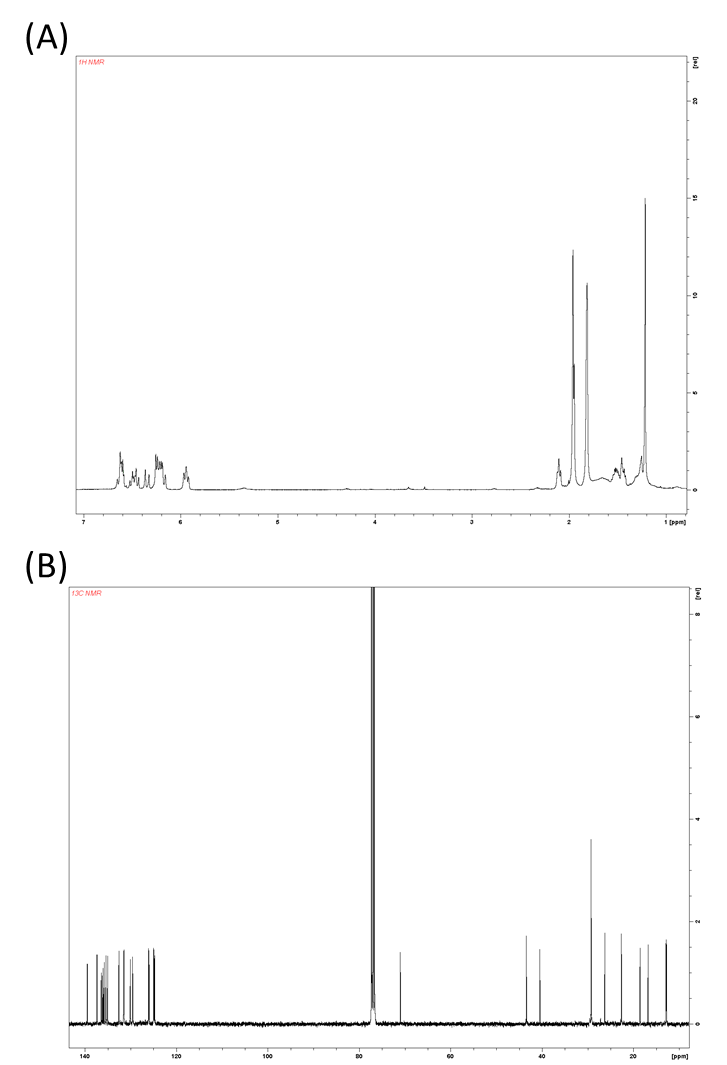


Fig. S2 NMR spectra of 5-hydroxy-5,6-dihydro-4,4'-diaponeurosporene (“**3**”) in CDCl_3_. (A) ^1^H NMR. (B) ^13^C NMR.


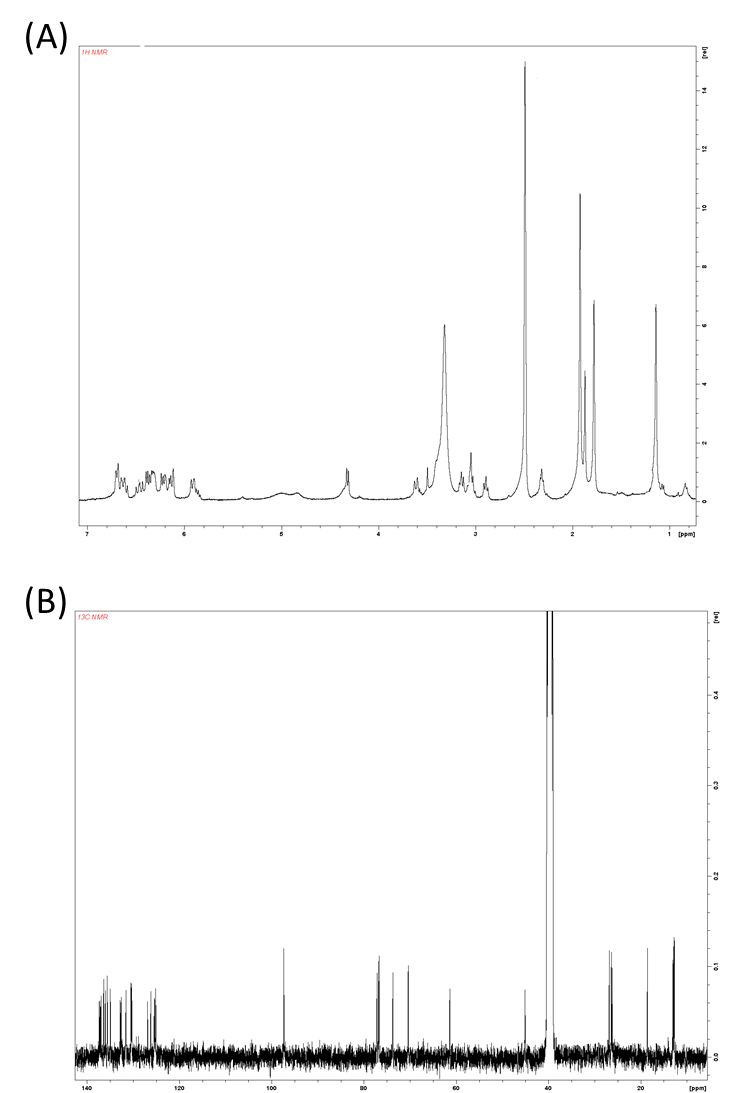


Fig. S3. NMR spectra of 5-glucosyl-5,6-dihydro-4,4'-diapolycopenene (“**5”**) in DMSO-*d*_6_. (A) ^1^H NMR. (B) ^13^C NMR.


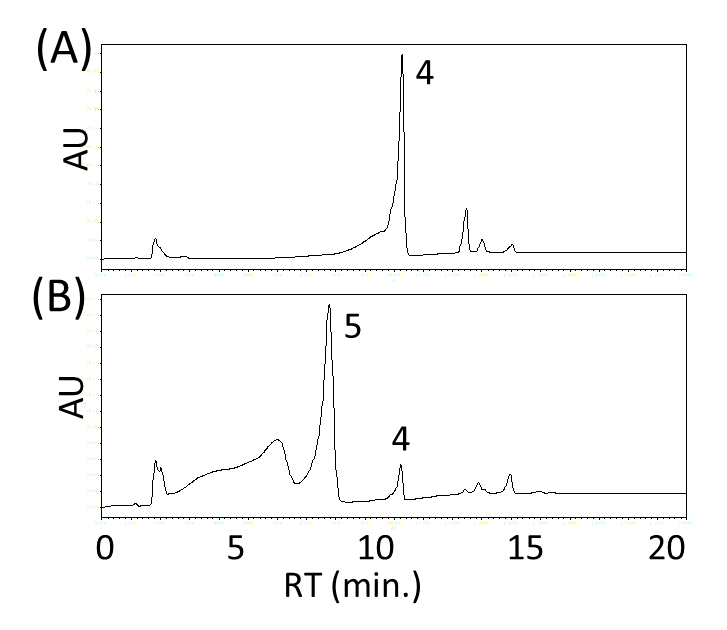


Fig. S4. Functional analysis of *orf2*.

HPLC chromatogram of the extracts of the *E. coli* expressing the plasmids pAC-HIMNFNb + pUC-Orf2 (A) and pAC-HIMNFNbO5 + pUC-Orf2 (B). 4; 5-hydroxy-5,6-dihydro-4,4'-diapolycopene, 5; 5-glucosyl-5,6-dihydro-4,4’-diapolycopene. In both cases, no new peak was found.

Table S1. Primers used in this study.

* The restriction sites are underlined. The SD sequences are bold.

Table S2. Accession numbers of the genes used in Fig. 2A.

| organism | gene | acc no. |
| --- | --- | --- |
| *Staphylococcus aureus* | *crtP* (*crtNb*) | WP_102782088.1 |
| *Staphylococcus aureus* | *crtN* (*crtNa*) | WP_057520765.1 |
| *Staphylococcus aureus* | *crtNc* | WP_001084326.1 |
| *Halobacillus halophilus* | *crtN* (*crtNa*) | ACM07424.1 |
| *Halobacillus halophilus* | *crtNb* | ACM07427.1 |
| *Halobacillus halophilus* | *crtNc* | ACM07425.1 |
| *Methylomonas* sp. strain 16a | *crtN* (*crtNa*) | AAX46183.1 |
| *Methylomonas* sp. strain 16a | *crtNb* | AAX46185.1 |
| *Methylomonas* sp. strain 16a | *aldH* (*crtNc*) | AAX46184.1 |
| *Rhodobacter capsulatus* | *crtI* | ADE84444.1 |
| *Pantoea ananatis* | *crtI* | AER34890 |
| *Lactobacillus plantarum* | *crtN* (*crtNa*) | [WP_011102097.1](https://www.ncbi.nlm.nih.gov/protein/WP_011102097.1) |
| *Bacillus infantis* | *crtNb* | AGX02538.1 |
| *Bacillus firmus* | *crtNc* | AGX02539.1 |
| *Bacillus firmus* | *crtN* (*crtNa*) | AGX02541.1 |

Table S3. Accession numbers of the genes used in Fig. 2B.

| organism | gene | acc no. |
| --- | --- | --- |
| *Bacillus horikoshii* | *crtM* | WP_148978645.1 |
| *Halobacillus halophilus* | *crtM* | WP_014643004.1 |
| *Lactobacillus plantarum* | *crtM* | WP_015826039.1 |
| *Staphylococcus aureus* | *crtM* | WP_060552587.1 |
| *Methylomonas methanica* | *crtM* | WP_013820065.1 |
| *Pantoea ananatis* | *crtB* | WP_105077524.1 |
| *Rhodobacter capsulatus* | *crtB* | WP_023911026 |
| *Synechocystis* sp. PCC 6803 | *PDS* | P37294.1 |
| *Nicotiana tabacum* | *PDS* | NP_001312950.1 |
